# A Novel Microcatheter Enters the Chronic Total Occlusion Space: An Advancement or Just Another Device?

**DOI:** 10.1016/j.jscai.2024.102144

**Published:** 2024-06-04

**Authors:** Eric Rothstein, Hannah Chaudry, Jesse Kane

**Affiliations:** a Dartmouth-Hitchcock Medical Center Heart and Vascular Center, Geisel School of Medicine, Lebanon, New Hampshire; b Department of Cardiology, University of Vermont Larner College of Medicine, Burlington, Vermont

**Keywords:** chronic total occlusion, complex high-risk indicated percutaneous coronary intervention, percutaneous coronary intervention

The publication of the BIOMICS study by Sidik et al^1^ is unlikely to be heralded as a major advance in the world of complex coronary intervention and chronic total occlusion (CTO) percutaneous coronary intervention (PCI); however, it still holds great meaning for the space. The BioMC microcatheter (Biosensors International) itself does not appear to be particularly extraordinary—another low profile lubricious pushable microcatheter. There are already multiple similar devices, such as the Finecross (Terumo Medical), Turnpike LP (Teleflex), and Caravel (Asahi Intecc) microcatheters (among others) available to operators. Based upon the results of this trial, the BioMC microcatheters do not appear to be a quantum leap forward in device technology, boasting a crossing profile and other deliverability characteristics similar to devices already on the market.

However, the publication of this trial is important in the CTO space because of what it means for the economics of accessibility in the field moving forward. CTO PCI is limited to a select few centers globally. Only a small minority of patients presenting with a CTO on angiography are even offered the option for CTO PCI and it is likely that ischemia secondary to a CTO remains undertreated.^2^ Although learning the various crossing techniques and skill sets may be daunting, the cost required to stock a CTO CART with various specialty wires, microcatheters, and other equipment represents another barrier to entry and adoption for many operators and cath labs, especially for those in less-resourced institutions. Additionally, even in larger institutions, procedural costs represent a significant challenge that hinders growth in many programs. Increased choices and the development of tools with cost effectiveness in mind will allow for competitive pricing. This will ideally increase availability and will help to reduce cost barriers for CTO PCI programs, ultimately improving accessibility for patients.

We commend the authors for their study design as they eloquently demonstrate how to study device-specific safety and procedural efficacy in CTO intervention. This type of device analysis lays the groundwork for comparative technical analyses of devices in future studies. Given the significant lesion variability and technical complexities of CTO PCI, coupled with the tremendous variability in equipment and techniques utilized by operators, it is incredibly challenging to make any comparisons at all. Specifically, there are 5 major CTO crossing algorithms^3^ (along with the algorithms within the algorithms), dozens of microcatheters, guide extensions, and wires with countless ways to use them in lesions that all seem to behave differently.^4^ However, there is a paucity of randomized trials that compare devices such as microcatheters head-to-head in CTO PCI subsets. Thus, as operators, we are typically left relying on anecdotal experience from our mentors, colleagues, and our own cases of what techniques and devices lead to success, failure, and complications. The BIOMICS study highlights key factors on how to study and evaluate devices, paving the way for what comparative device studies in our field could look like. Specifically, the evaluation of single initial device crossing success in addition to procedural success may represent a specific end point to be evaluated by other devices or crossing techniques in future trials. Ideally, these future trials could help streamline and simplify decision-making in this field faced by ever-growing complexity, and the BIOMICS study can serve as a template for design of these studies.

In summary, although the BioMC microcatheters are unlikely to make major strides toward improving CTO PCI success rates or procedural safety, the notion of an economical device is key to improving CTO accessibility in the future. Through their study design, the authors demonstrate an effective way to evaluate devices that can guide us on a path for investigator-initiated comparative device trials in the future.
